# Patient and family engagement in the development of core outcome sets for two rare chronic diseases in children

**DOI:** 10.1186/s40900-021-00304-y

**Published:** 2021-09-14

**Authors:** Shelley M. Vanderhout, Maureen Smith, Nicole Pallone, Kylie Tingley, Michael Pugliese, Pranesh Chakraborty, Sylvia Stockler, Martin Offringa, Nancy Butcher, Stuart G. Nicholls, Beth K. Potter

**Affiliations:** 1 Clinical Epidemiology Program, Ottawa Hospital Research Institute, 501 Smyth Road, Box 201B, Ottawa, ON K1H 8L6 Canada; 2 School of Epidemiology and Public Health, University of Ottawa, 600 Peter Morand Crescent, Room 101, Ottawa, ON K1G 5Z3 Canada; 3 Patient Partner, Canadian Organization for Rare Disorders, Toronto, ON Canada; 4 Patient Partner, Director of CanPKU and Parent of a Child with an Inherited Metabolic Disease, Toronto, ON Canada; 5 Newborn Screening Ontario, Children’s Hospital of Eastern Ontario, 415 Smyth Road, Ottawa, ON K1H 8M8 Canada; 6 Division of Biochemical Genetics, BC Children’s Hospital, Department of Pediatrics, University of British Columbia, 4480 Oak Street, Vancouver, BC V6H 3V4 Canada; 7 The Hospital for Sick Children Research Institute, Peter Gilgan Centre for Research and Learning, 686 Bay Street, 11th Floor, South 16, Toronto, ON M5G 0A4 Canada; 8 Department of Psychiatry, Faculty of Medicine, University of Toronto, 250 College St, 8th Floor, Toronto, ON M5T 1R8 Canada

**Keywords:** Core outcome sets, Medium-chain acyl-CoA dehydrogenase deficiency, Phenylketonuria, Patient engagement, Consensus

## Abstract

**Background:**

Core outcome sets (COS) are lists of consensus-determined outcomes to be measured and reported in all clinical research studies within a disease area. While including patients and families in COS development to improve their relevance and applicability to patient values is key, there is limited literature documenting practical barriers and facilitators to successful patient engagement in COS development. In this paper, as researchers and patient partners, we provide a resource for COS developers to meaningfully and effectively engage patients and families.

**Main body:**

To establish a consensus-based COS for children with two inherited metabolic diseases (medium-chain acyl-CoA dehydrogenase deficiency and phenylketonuria), we conducted an evidence review, Delphi survey, and workshop. Two adult patient partner co-investigators co-developed the study protocol, co-designed strategies to address challenges with incorporating patient perspectives, and led all patient engagement activities, including communication with a group of family advisors. Seven adult family advisors received training about COS development and subsequently contributed to Delphi survey development, outcome definitions, the consensus workshop, and selection of outcome measurement instruments. Patient partner co-investigators and family advisors were essential to the successful design, conduct, and completion of the two COS. Patient partner co-investigators supported the understanding, inclusion and engagement of family advisors, and helped develop accessible tools to determine patient-oriented outcome measurement instruments. Patient partner co-investigators and family advisors collaborated with the study team to co-develop surveys, modify technical language, and recruit participants to the study. Together, we addressed challenges to patient engagement in COS development such as unfamiliarity with study methods, comprehensibility of materials and ongoing engagement, and power imbalances between team members.

**Conclusion:**

Our approach to patient and family engagement in COS development for two rare conditions for children was feasible and considered valuable by all study team members, including patients and family members, in improving the relevance of the deliverable to patients. This approach to patient engagement in developing COS can be applied to other paediatric disease contexts, allowing patient and family perspectives to influence the direction of future studies to develop COS.

**Supplementary Information:**

The online version contains supplementary material available at 10.1186/s40900-021-00304-y.

## Plain English summary

Core outcome sets (COS) are lists of outcomes agreed upon by a group of people to be measured and reported in studies about certain diseases and populations. Core outcomes are meant to represent what is useful to study from the perspectives of health care providers, researchers and patients. For researchers who seek to include patients in the development of a COS, there is little guidance about how to do this well. We recently developed COS for two rare diseases in children, medium-chain acyl-CoA dehydrogenase deficiency and phenylketonuria. We did this by reviewing available information from published research, surveying health care providers, researchers, and patients, and eventually coming to agreement during a workshop. We included two adult patient partner co-researchers who helped design the COS study and co-developed the patient engagement strategy. These partners formed relationships with seven adult family advisors, who helped ensure that materials were accessible, participated in outcome selection, and helped select tools to measure core outcomes. Strategies we used to engage patient partners included a) training about both the scientific research process and how to help other researchers in the future, and b) frequent communication about study progress and how family advisor feedback was used. Also, we made sure that the impacts of power imbalances between health care providers, researchers and patients were low. Our approach to patient engagement in COS development for two rare conditions in children proved to be both feasible and considered valuable by all study team members, including patient partners and family advisors. To include patient perspectives and values, future COS developers may take a similar approach.

**Supplementary Information:**

The online version contains supplementary material available at 10.1186/s40900-021-00304-y.

## Background

Core outcome sets (COS) are standardized lists of outcomes determined by consensus, as a minimum set, to be measured and reported in studies within a disease area [1]. Each outcome is intended to be measured by a suitable Outcome Measurement Instrument (OMI). Researchers are increasingly including patient partners in COS development to improve the relevance and applicability of research to patient values and clinical care [2, 3]. COS have value for research on rare diseases due to limited patient populations and replication in research [4, 5]. Collaborating with patient partners with lived experience of rare disease in their children is especially important due to heterogeneity in disease presentation, progression, and therapy, small patient and clinician communities, relatively frequent opportunities to participate in research regarding interventions that are not supported by rigorous evidence (given small numbers of patients available for studies), increased feeling of pressure to participate in research to improve symptoms or advance knowledge about rare diseases, few standards of care, and varied access to treatment [6]. The COMET (Core Outcome Measures in Effectiveness Trials) [1] Initiative has provided guidelines for the development of COS, including patient engagement, where researchers are encouraged to include patients with experience of the studied condition as members of the research team to ensure COS are relevant and their development is trustworthy to patients [7]. Despite this available guidance, there is limited literature documenting barriers and facilitators to patient and public engagement in COS development [8], and particularly in the context of rare diseases.

We recently developed the first COS for two rare inherited metabolic diseases (IMDs) in children, medium-chain acyl-CoA dehydrogenase (MCAD) deficiency and phenylketonuria (PKU) [9–11], following the approach recommended by COMET [1]. We found that parent and caregiver participation in the design and conduct process was paramount to capturing patient-important outcomes in both COS for MCAD deficiency and PKU, and also offered key insights which may be transferrable for patient engagement in the development of other COS and future research activities. In this article, we describe our experience in partnering with patients and caregivers in COS development, identify aspects that worked well, as well as those which proved more challenging while engaging patients during this process, and reflect on how to address and overcome similar challenges going forward. This information may serve as a resource to COS developers for engaging in effective, meaningful patient partnerships.

## Main text

### COS development study

**Fig. 1:**
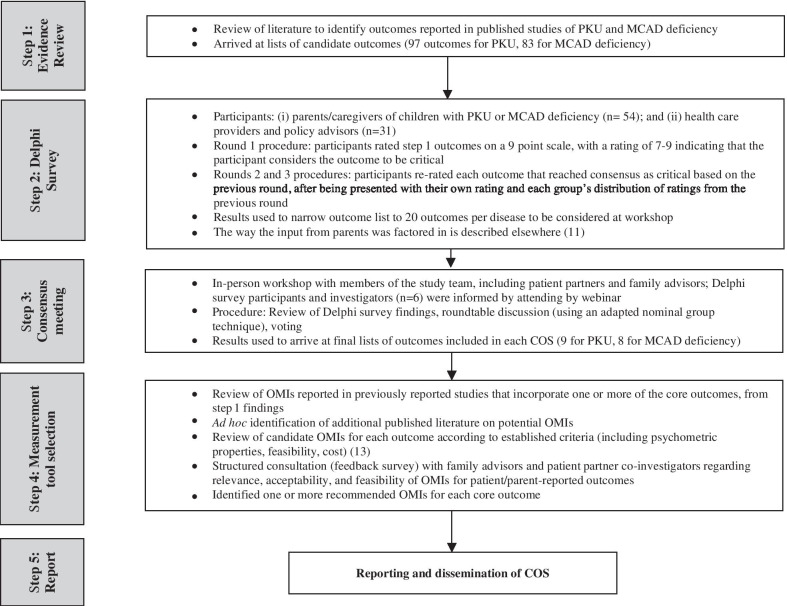
Outline of core outcome set (COS) development process [9, 11]

The protocol for the development of COS for MCAD deficiency and PKU has been published elsewhere [10] and results of this process have been published [9, 11]. Briefly (see overview, Fig. 1), the study included an evidence review, Delphi survey, and workshop to arrive at a consensus-based COS for each disease [12]; and a further consultation to select recommended OMIs for each outcome included in the COS. Here we describe the methods specific to patient and family engagement, which were guided by COMET [7].

### Recruitment and overall roles of patients or their family members as partners and advisors

The research team recognized the importance of having patient partners with experience in research who could contribute at an investigator level and lead a patient engagement strategy; and including other family advisors who may not have had prior research experience but wanted to contribute and had valuable lived experience. Including patient partner co-investigators and family advisors was important to allow for varied levels of engagement across the International Association of Public Participation (IAP2) [13] spectrum. This resulted in co-investigators engaged throughout the entire process and opportunities for parents who were new to patient engagement to advise at key points. This also served to build capacity in patient and family engagement in our research program. Our patient engagement strategy was informed by exemplar studies/frameworks such as the Canadian Institutes of Health Research (CIHR) Strategy for Patient Oriented Research (SPOR) [14] and Ladders of Citizen Participation [15, 16].

#### Patient partners

Two patient partner co-investigators (MS, NP), who were both known to the study team as members of the rare disease community and experienced patient partners, were recruited at the grant application stage and were actively engaged during the entire study. One patient partner was a parent of a child with PKU and board member for a PKU patient advocacy group and the other was a patient with a rare disease who had a long history of engagement in the health care system and was on the executive of the Canadian Organization for Rare Disorders. Together, they co-led the patient engagement strategy along with the principal investigator (BP). Roles of the patient partner co-investigators included contributing to study protocol development, identifying challenges to incorporating patient perspectives and designing strategies to address those challenges, leading patient engagement activities such as newsletters and training, and recruiting and communicating with family advisors. As part of the study’s knowledge translation strategy, patient partners also co-developed and presented on the patient engagement approach at several conferences [17–23].

#### Family advisors

Seven parents of children with IMDs (including but not limited to PKU and MCAD deficiency) were recruited by metabolic clinician investigators (PC, SS) at two centres (Ottawa and Vancouver) to form a Family Advisory Forum (FAF). FAF members provided input at key points during the study including development of the Delphi survey instructions and content, revision of lay definitions of candidate outcomes, contributing to outcome selection at the consensus workshop, and contributing to the selection of outcome measurement instruments.

Despite our efforts to reach a broad range of families, FAF members were individuals who were able (from an educational, economic, and self/child wellness perspective) to devote considerable time to the study. In future, we would like to develop strategies to foster greater diversity among patient and family advisors, including inviting youth with lived experience of IMDs to participate.

With respect to honoraria, patient partner and FAF member compensation was planned for in the grant application stage of the COS development study, and provided annually by cheque or gift card in accordance with the Canadian Institutes of Health Research SPOR guidelines for compensation [24].

To incorporate the perspectives of a broad group of family members in the development of each COS, the Delphi surveys also included parents of children with MCAD deficiency or PKU as study participants (Fig. 1). These parent participants also had the option to attend and observe the consensus workshop online via webinar. While the input of parent participants in the Delphi surveys was central to the development of the COS, our focus here is on patient engagement (patients or family members in co-investigator or advisory roles). The remainder of this paper thus focuses on the roles of the two patient partner co-investigators (MS, NP) and the 7 family advisors, as described above.

### Training of family advisors at the start of the study

From the outset it was recognized that FAF members may not have participated as advisors in research before; further, the purpose of this study (developing a COS to inform future research) was likely distant from the immediate concerns of families. Our solution to this challenge was to develop and deliver an in-person training workshop at the beginning of the study. This training, developed and led by the patient partner co-investigators (MS, NP) and principal investigator (BP), incorporated Strategy for Patient-Oriented Research (SPOR) [25] and COMET [1, 26] principles and included material adapted from both initiatives (see supplementary information in Additional File 1). The focus of the training was to understand the phases of COS development and how patient perspectives were vital to selecting patient- and family-relevant outcomes for use in future studies. FAF members were provided information about the purpose of COS development and importance of patient engagement, methods for the study (aligned with Fig. 1), and expectations for working together. These expectations included anticipated time commitments and study timelines, honoraria provided in appreciation for FAF members’ time, and the nature of their advisory role, including a flexible approach which recognized FAF members’ other commitments and priorities.

We believe that this in-person training (which could be adapted to be delivered online) contributed greatly to FAF members’ understanding of the study and resulted in enthusiasm to undertake the study together. For example, one FAF member disclosed that, although information about the study had been sent in advance, it was only at the in-person training that they had fully understood the purpose and methods of the research and their role. From the perspective of the patient partners and principal investigator, establishing relationships with FAF members during training was very helpful to open lines of communication, create an atmosphere of sharing and familiarity with one another, minimize power imbalances, and promote mutual learning.

### Ongoing engagement with the FAF

Maintaining the interest of FAF members throughout the research project (2 + years) was challenging, given that there were sometimes long stretches with little to report (e.g., during Step 1: review of published evidence). This has been identified by other researchers who engage patients [27, 28]. The patient partner investigators wrote newsletters to keep in touch with FAF members throughout the project to highlight progress, communicate new developments, demonstrate how their feedback was used, maintain relationships with the FAF, and check on continuing interest in contributing to the study (see supplementary information in Additional File 1). The newsletters included a “Getting to Know You” section where families and members of the research team could introduce themselves if they wished. FAF members often responded to the newsletter to say that they were glad to be kept up to date and to know they hadn’t missed anything, underscoring the importance of establishing relationships with patient partners through frequent and consistent communication.

### Role of patient partners and family advisors in the Delphi surveys

Disease-specific online Delphi surveys were adapted from materials available from the COMET Initiative [1] and co-drafted by the patient partners and principal investigators. As the study topic was probably unfamiliar to most parent participants in the Delphi survey, we sought additional critical input from FAF members to ensure that the online survey materials were accessible. Specifically, collaborating with the research team by email and sometimes using web surveys or telephone calls, the FAF members reviewed study invitations and information to ensure that the language used was clear and understandable by parent participants. For example, information provided to parent participants prior to completing the survey included answers to questions such as “What are core outcome sets?”, “Why do we want to incorporate the opinions of families in developing a COS for PKU and for MCAD deficiency?” and “What is a Delphi survey?”. FAF members and patient partners also edited definitions of the outcomes presented within the surveys to ensure broad accessibility, which is recommended by Core Outcome Sets Standards for Development (COS-STAD) [29]. Feedback from the FAF members resulted in substantial changes to the presentation of the Delphi survey and to the definitions of the outcomes. These changes were communicated back to FAF members so that they could readily identify that their feedback was valuable.

Based on feedback we received from study investigators, parent partners, and participants, in the future we would streamline our approach to creating Delphi surveys. Though many outcomes were identified in the literature searches, it was clear that some would not be included in the final COS. In an effort to lessen the time commitment required of our parent participants, we would narrow the selection criteria for outcomes to be considered in Delphi surveys by excluding those which are clearly outside the scope of the COS upfront [30].

### Role of patient partners and family advisors in the in-person consensus workshop

A team of 12 clinicians, methodologists and health system investigators, two patient partners and four FAF members (the remaining three were invited but were unavailable to attend) met in person to reach final consensus on the outcomes to be included in each disease-specific COS based on Delphi survey results (Fig. 1). It was important to us that FAF members were supported to participate fully and meaningfully in the consensus workshop. We were concerned about potential power imbalances in a meeting that included FAF members alongside clinician investigators and methodologists, which could be exacerbated by the fact that for some FAF members, their child’s physician would be in attendance. This is common in the rare disease community, where patient and clinician groups tend to be small. The patient partner co-investigators, principal investigator, and a clinician investigator met during the workshop planning stage to discuss and develop strategies to address these issues. Prior to the meeting, these strategies included the development of pre-workshop materials that were circulated to the FAF in advance to explain the details of the workshop (a summary of study results to date, information about what to expect at the workshop, and a list of workshop attendees); and an in-person pre-meeting, which included study investigators, both patient partner co-investigators, and the workshop chair, for FAF members to meet the workshop chair and review workshop materials and procedures in advance. During the in-person meeting, strategies to support participation by FAF members included a presentation led by one of the patient partner co-investigators to communicate the value of the lived experiences of patients and parents in developing each COS and to describe how the FAF members were involved in the study; including a workshop chair who had experience with patient engagement; strategic seating and name cards so that FAF members were seated among other workshop participants rather than in a separate cluster; and use of a modified Nominal Group Technique during the discussion [31], as has been suggested by COMET [1, 32, 33]. Specifically, the Nominal Group Technique, whereby everyone was provided one minute to share suggestions for the three most important outcomes during the workshop discussion, was intentionally chosen to convey the equal importance of each attendee’s opinion. Of these strategies, use of the Nominal Group Technique and the pre-workshop training co-led by patient partner co-investigators seemed to be particularly effective.

Delphi survey participants who wished to attend the consensus workshop were able to join by webinar; as this stage was not a part of the research process, this option was intended to be informative only.

Workshop attendees returned a brief exit survey at the end of the workshop. The results indicated that all participants, including FAF members, had a highly positive experience, which instilled confidence in our choice of workshop methodology and the quality and quantity of pre-workshop training. For example, on a follow-up survey, all FAF members agreed or strongly agreed that the pre-meeting was helpful, they were able to express their views freely, and their input was considered during the discussion. One FAF member indicated that they would have preferred some additional pre-meeting preparation by telephone. In the future, we would include a standardized tool such as the GRIPP2 checklist to ensure comprehensive assessment and reporting of patient engagement methods across the study [34].

### Role of patient partners and family advisors in outcome measurement instrument selection

We used a literature search to identify potential OMIs for each of the outcomes in each COS, and documented the reliability, validity, cost, and considerations of feasibility (e.g., language availability, mode of administration) for each candidate OMI (Fig. 1). For outcomes deemed best measured by patient/parent self-report, and as part of an integrated knowledge translation project regarding COS implementation, we invited FAF members to provide feedback on the OMIs. The patient partner co-investigators collaborated with the research team to develop a short feedback survey that was sent to interested FAF members along with sample versions and brief descriptions of each OMI, and a guide to explain how to review an OMI. When reviewing each patient/parent self-reported OMI, FAF members were asked to comment on relevance, alignment with the outcome definition, perceived feasibility for parent/caregiver reporting, and acceptability. FAF members were also asked to select the most appropriate instrument where more than one option existed. They could submit feedback via email, telephone, or in-person at a knowledge translation meeting. The feedback was used to select the final recommended patient/parent reported OMIs, with family advisors raising issues connected to both relevance and acceptability/sensitivity regarding the content of the OMIs.

## Conclusion

Our approach to engage parents and caregivers in COS development had some key strengths. Parents and patients with both lived experience in rare diseases and in research were part of the team of study investigators and co-designed a patient engagement strategy which aimed to gain important family perspectives. These patient partner co-investigators collaborated with the team to co-design the patient engagement strategy and were empowered to take on leadership roles and responsibilities. For example, when patient partners led training workshops for FAF members, power dynamics that may have existed if a clinician or researcher were leading were minimized, which may have allowed FAF members to feel comfortable asking more questions and prepared them to engage fully in the consensus workshop. The extensive training and support provided by patient partners to FAF members promoted an inclusive, neutral environment. Recommendations for patient engagement in COS development given by COMET [7] formed the framework for our methods; additional strategies which we found important were maintaining relationships with family advisors during periods of low study activity and taking intentional steps to minimize power imbalances within the study team. Several strategies were adopted to mitigate challenges that were anticipated at each stage of the study. Feedback from the FAF and their engagement throughout suggested that these strategies were critical to the success of the study. Finally, the capacity building that occurred has been beneficial to our ongoing research. The two patient partners were offered training opportunities and benefited from presenting and attending conferences. They have both continued being engaged with our research team. Some members of the FAF have continued to be engaged in our studies.

Our method also had limitations. Including parents as research co-investigators and advisors allowed us to understand outcome priorities from family-centred perspectives, given that these COS were focused on children under age 12 years. Yet, lack of inclusion of youth in our COS development process limited our ability to incorporate youth views and preferences. This is a key priority for future research, ensuring that COSs reflect the perspectives of individuals of all ages who have lived experience with the studied condition. This is particularly important given that prior studies have found that youth and adults, including parents, may have different outcome preferences [35–38]. Engaging youth in health research can empower young patients, offer opportunities to learn more about their health, and participate in reciprocal learning, where youth share their lived experience to advance research [36]. While some materials and guidelines for patient engagement in COS are available, clear and evidence-based instruction is lacking which meant improvisation was occasionally necessary. However, this allowed our approach to be responsive to our unique setting, research team and participants and we are now able to share insights from our approach to inform future similar studies. Future work to collate individual study experiences may be useful for identifying approaches that would be universally helpful. In addition, the diversity among patient and family partners and advisors could still be improved. Recruitment of patient and family partners was conducted via clinician referral. In future projects, we would use a more systematic approach to include a broader sample of partners to ensure representation and diversity. Though we sought continuous informal feedback about our approach from the study team, including patient partners and family advisors, we did not use a formal evaluation tool in this exercise, which may have provided us structured information about how to improve our methods throughout the study and in the future.

## Supplementary Information

**Additional file 1**. Newsletter excerpts and training materials.

## Data Availability

Data sharing is not applicable to this article as no datasets were generated or analysed during the current study.

## Abbreviations

CIHR: Canadian Institutes of Health Research
COMET: Core Outcome Measures in Effectiveness Trials
COS: Core outcome set
COS-STAD: Core Outcome Sets Standards for Development
FAF: Family Advisory Forum
IMD: Inherited metabolic disease
MCAD: Medium-chain acyl-CoA dehydrogenase
OMI: Outcome measurement instrument
OSSU: Ontario Strategy for Patient Oriented Research Support Unit
PKU: Phenylketonuria
SPOR: Strategy for Patient Oriented Research
