# Supplementary material for: Patient and family engagement in the development of core outcome sets for two rare chronic diseases in children
Source: Res Involv Engagem. 2021 Sep 14;7:66. doi: 10.1186/s40900-021-00304-y (PMC8439069; doi:10.1186/s40900-021-00304-y)
Supplement: Supplementary file 1 — Additional file 1. Newsletter excerpts and training materials. [file 40900_2021_304_MOESM1_ESM.pdf]

Excerpts from Family Advisory Forum Update Newsletters

The patient partner co-investigators designed 7 newsletters over the course of two years to update members of the FAF. These updates included a training component.

Update #1

Feedback on Recruitment Materials

Thanks to all members of the Family Advisory Forum who provided such insightful and useful feedback on our recruitment materials. There were many suggestions on how to improve the telephone script and we have made several changes. In some sites this is the only means of initially contacting families, therefore, this script is of vital importance. Your feedback on all four documents was most helpful.

Did you know?

Response rate - 100%
Average time to complete survey – 31 minutes
Number of changes made to documents – 22
Impact – Immeasurable!

Update #2

Research Update

Where are we at the end of Year One?
What is causing all the delays?

We are ready to refine the definitions of 98 core outcomes for PKU and 84 for MCAD Deficiency, which brings us one step closer to launching the Delphi Survey.

Next Steps

1) Launch of first round of Delphi survey in early September with two weeks response time
2) Round two of Delphi Survey to build consensus
3) Round three – if necessary
4) Analysis of Delphi survey results by the research team – late October
5) Planning of face-to-face meeting in November

Now for the delays … You're probably thinking that you wouldn't be doing so well at your day job if your timelines were that far off! Well, in the world of academic research, estimates are best guesses and it's often par for the course that projects go on for much longer than anticipated.

Why is it so difficult to come up with an accurate timeline?

- It's difficult to know how many articles will surface during the literature survey. In our case, it was overwhelming and we spent much a great deal of time on this aspect.
- There were more outcomes than we anticipated.
- It's difficult to predict how many edits will be required from the different Research Ethics Boards.

Update #3

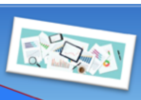

What are outcome measurement instruments?

Examples of outcome measurement instruments for PKU and MCADD

- Quality of life questionnaires (both PKU and MCADD)
- Methods and frequency of measuring and reporting blood phenylalanine (PKU)
- Criteria for defining and documenting metabolic decompensation (MCADD)

The outcomes in the core outcome set are WHAT should be measured and reported in clinical trials, whereas the **outcome measurement instruments** are HOW these outcomes should be measured.

- The literature survey that was completed before the Delphi survey identified outcomes as well as the existing measurement instruments.
- Now it is time to select the best outcome measurement instruments. Many factors such as the type, ease of administration, costs, patients' and clinicians' understanding of the tool, and length and completion time are taken into consideration.

Update #4

Why are patient reported outcomes (PROs) so important in your child's care and in research?

PROs are important because they provide a patient and/or caregiver perspective on a disease/treatment that might not be captured by a clinical measurement (e.g. a blood test, ultrasound, etc.) but may be as important to the patient and their adherence to the treatment (the degree to which the patient correctly follows medical advice).

Why is your perspective of vital importance?

PROs must be carefully defined so that they capture information that is important to patients. For this study, we need your perspectives on measurement tools: do they respond to your needs and are they doable given the high burden of caring for a child with an IMD. We also want to hear your perspectives on how the data is collected and used, including in registries.

What does this have to do with core outcome sets?

Providing recommendations regarding how outcomes should be measured, including specific outcome measurement instruments, is part of developing a core outcome set. If researchers can agree on standard tools it will be easier to make comparisons across studies and combine data.

Family Advisory Forum

Project to develop core outcomes for inherited metabolic diseases

Introductory Meeting

July 10, 2017

uOttawa

CIMDRN
RCRMMH

CIHR IRSC
Canadian Institutes of Health Research
Instituts de recherche en santé du Canada

OBJECTIVES

- Meet each other - members of the Family Advisory Forum and research team
- Overview of the research project
- Discuss expectations of Family Advisory Forum members
- Gain insight into *why* you are here
- Recognize the power of your engagement in core outcome sets

WELCOME

- Introduction of research team members
- Introduction of Patient Advisory Forum members

WHY A FAMILY ADVISORY FORUM?

- To provide an opportunity for the study team to learn the disease expertise and the insights patients and families have gained from their experiences with IMDs
- To ensure that the final outcomes developed are important and meaningful to patients/ families as well as researchers
- To assure inclusion of the perspectives of patients and families across all areas of the project

PATIENT ENGAGEMENT

As partners in research, what do patients and caregivers bring to the table?

- Passion
- Expertise
- Credibility
- Patient/Caregiver perspective

WHAT DO WE MEAN BY PATIENT ENGAGEMENT IN RESEARCH?

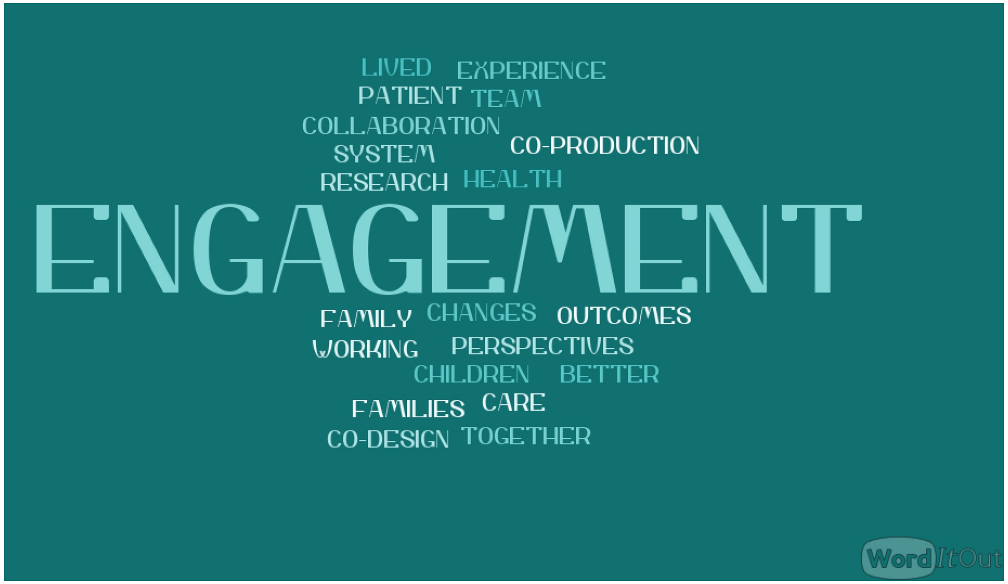

"Meaningful and active collaboration in governance, priority setting, conducting research and knowledge translation."

- Canadian Institutes of Health Research (CIHR) Strategy for Patient-Oriented Research (SPOR)

From: SPOR Patient Engagement Framework: https://cihr-irsc.gc.ca/e/documents/spor_framework-en.pdf

PATIENT-ORIENTED RESEARCH GUIDING PRINCIPLE # 1

Inclusiveness

"Patient engagement in research integrates a diversity of patient perspectives and research is reflective of their contribution – i.e. patients bring their lives into this"

– CIHR SPOR

From: SPOR Patient Engagement Framework: https://cihr-irsc.gc.ca/e/documents/spor_framework-en.pdf

GUIDING PRINCIPLE # 2

Support

"Adequate support and flexibility are provided to patient partners to ensure that they can contribute fully to discussions and decisions. This implies creating safe environments that promote honest interactions, cultural competence, training, and education. Support also implies financial compensation for their involvement."

– CIHR SPOR

From: Strategy for Patient-Oriented Research (SPOR) Patient Engagement Framework: https://cihr-irsc.gc.ca/e/documents/spor_framework-en.pdf

GUIDING PRINCIPLE # 3

Mutual Respect

"Researchers, practitioners and patient partners acknowledge and value each other's expertise and experiential knowledge."

– CIHR SPOR

From: SPOR Patient Engagement Framework: https://cihr-irsc.gc.ca/e/documents/spor_framework-en.pdf

GUIDING PRINCIPLE # 4

Co-Build

“Patient partners, researchers and practitioners work together from the beginning to identify problems and gaps, set priorities for research and work together to produce and implement solutions.”

- CIHR SPOR

From: SPOR Patient Engagement Framework: https://cihr-irsc.gc.ca/e/documents/spor_framework-en.pdf

CO-LEARNING

- The goal is not to turn patients and caregivers into researchers, but to help them understand and be partners in the research process.
- Health care providers and researchers will also be learning – about patient-centredness and what it's like to live with a disease.

Adapted from: SPOR Patient Engagement Framework: https://cihr-irsc.gc.ca/e/documents/spor_framework-en.pdf

OUR STUDY

- Purpose: to facilitate research that will tell us which treatments are most effective for children with inherited metabolic diseases
  - Initial focus is on PKU and MCAD deficiency
- We will do this by establishing agreed core outcome sets for these conditions
- If these outcomes are routinely integrated into health care and research, it will be easier to evaluate existing and new treatments

REGISTRY-BASED RESEARCH

- We have a research network across 13 Canadian hospitals that provide treatment to children with inherited metabolic diseases
- In the future, we hope to invite the families who participate in this network to become part of a consent-based registry
- The outcomes that we develop in this project would then be collected as part of that registry
- New clinical trials and other studies could then be conducted more easily

CIMDRN
Canadian Inherited Metabolic Diseases Research Network

WHAT IS A REGISTRY?

- A database where individuals with a specific disease have consented to enter information about their health
- A way to collect research data about patients, such as diagnosis and important outcomes, in order to understand care and disease patterns
- We plan to use a registry to identify patients to invite into new studies
- The outcomes we collect will also be used in those new studies to evaluate treatments

WHAT ARE CORE OUTCOME SETS?

How health care treatments are developed

- Why do researchers measure ‘outcomes’
- For children with inherited metabolic diseases such as PKU and MCAD deficiency, outcomes might include:
  - Patient- or family-reported outcomes like quality of life
  - Clinical outcomes like growth or cognitive development
  - “Biomarker” outcomes like blood test results

WHAT ARE THE CHALLENGES IN MEASURING OUTCOMES?

- Measuring different outcomes = unable
  to compare or combine results
- Study team may only publish results of some outcomes, therefore, other researchers don’t have the full results of the study
- Rare diseases: we need to work together across clinical centres to conduct research on what treatments are most effective; difficult to do if we are all measuring different outcomes

HOW CAN WE SOLVE THIS PROBLEM?

- Agree on a set of main outcomes – a ‘core outcome set’ (COS)
- We can bring together different studies to get a better understanding of which treatments are most effective
- We can make best use of resources when we design new studies….

WHY IS IT SO IMPORTANT TO INVOLVE PATIENTS?

- Core outcome sets need to include outcomes that are **most** relevant to patients and caregivers
- Best way to do this is to involve patients and caregivers in their development
- There are many examples of outcomes that had been overlooked due to lack of patient involvement

COMET Initiative

https://www.comet-initiative.org/

COS SUCCESS STORIES

- Patients who participated in determining core outcome sets for rheumatoid arthritis determined that fatigue was an important outcome that should be measured in future studies

HOW ARE CORE OUTCOMES AGREED UPON

- Must ensure that the expertise of patients, caregivers, and professionals is used to develop core outcomes that are relevant to patients, caregivers and health professionals
- Often done through consensus methods

WHAT ARE CONSENSUS METHODS?

- Surveys, meetings, and discussions where the opinions of relevant experts are drawn together
- Patients and caregivers were not always involved in this type of work but the importance of including their first-hand experience of living with a condition is now recognized

HOW WILL WE DEVELOP CORE OUTCOME SETS?

- Our study has four parts:
  - A review of the literature to identify possible outcomes that might be relevant for PKU and MCAD deficiency (this is underway)
  - A set of consensus surveys ("Delphi surveys") to find out which of these outcomes are most important from the perspectives of patients or their caregivers and providers
  - A discussion of the findings to establish the core outcomes and to decide how best to measure them
  - A pilot test of the outcomes

DELPHI PROCESS SUMMARY

A list of outcomes is produced by the research team and sent to each patient and health professional to score the importance of each one

Round 1

Round 1 results are summarized and sent back to each person, together with a reminder of the person's own score for each outcome. Each person is asked to think about the group's results and decide if they want to change their score

Round 2

The process of seeing the results and re-scoring the outcomes can be repeated in a 3rd round

Further rounds

Face-to-face meeting with study team (including Family Advisory Forum) to discuss the results

Report produced identifying agreed outcomes of importance (core outcomes)

Adapted from: Young and Bagley. Including patients in core outcome set development: Issues to consider based on three workshops with around 100 international delegates. Research Involvement and Engagement. 2016;2:25.

ESTABLISHING OUTCOMES

- We will hold a face-to-face team meeting to discuss the final results of the Delphi surveys
- This meeting will include Family Advisory Forum members to ensure that the patient and family perspective is fully considered
- Once we have agreed on core outcomes, we will also discuss measures of the outcomes

ARE ALL OUTCOMES INCLUDED IN THE COS?

- Not all the outcomes endorsed or suggested by Delphi participants can be taken forward in the core outcome set, and while they may be very important, we have to make decisions to take only a few forward
- This doesn’t mean that the others are not considered, but in order to make sure that people do use the 'core' set, they have to balance importance with feasibility.

WHAT WILL I BE EXPECTED TO DO?

- Receive training in working with research teams on patient-oriented research
- Receive training to learn about core outcome sets and Delphi surveys
- Participate in the design of meaningful and user-friendly surveys
- Participate in the selection of patient/family-reported measures of outcomes

Life happens – let us know how we can support your continued involvement

GROUND RULES FOR EFFECTIVE COMMUNICATION

- Everyone’s opinion is listened to and respected equally
- All questions are good questions
- Respect the privacy of others in the group

MEETING SCHEDULE

- Teleconferences, approximately every 2 months
- In-person study team meeting (tentatively January 2018)
- Time investment: an average of 3 hours per month

REIMBURSEMENT

- We will reimburse you for any expenses you incur for the study (for example: for travel to meetings, we will reimburse costs of transportation, accommodation, meals)
- We can provide a travel advance to cover expenses up front, and will then reconcile based on actual receipts that you provide
- We will also provide a $500 honorarium – we will be in touch re: your preferences (e.g., we can provide up front or in installments)

NEXT STEPS

- We will send a project update by email, mid to late summer
- We will be in touch soon to set up teleconference dates
- Our next teleconference will be held in September to discuss the survey

We look forward to working with you!
